# Prognostic Value of Separate Extramural Vascular Invasion Reporting in Operative Samples of Rectal Cancer: Single-Institutional Experience

**DOI:** 10.3390/cancers16213579

**Published:** 2024-10-24

**Authors:** Mladen Djuric, Bojana Kožik, Tijana Vasiljevic, Aleksandar Djermanovic, Nevena Stanulovic, Marina Djuric

**Affiliations:** 1 Faculty of Medicine, University of Novi Sad, 21137 Novi Sad, Serbia; mladen.djuric@mf.uns.ac.rs (M.D.); tijana.vasiljevic@mf.uns.ac.rs (T.V.); nevena.stanulovic@mf.uns.ac.rs (N.S.); 2 Department of Surgical Oncology, Oncology Institute of Vojvodina, 21204 Sremska Kamenica, Serbia; djermanovic.aleksandar@onk.ns.ac.rs; 3 Laboratory for Radiobiology and Molecular Genetics, Vinča Institute of Nuclear Sciences, National Institute of Republic of Serbia, University of Belgrade, 11000 Belgrade, Serbia; 4 Department of Pathology and Laboratory Diagnostic, Oncology Institute of Vojvodina, 21204 Sremska Kamenica, Serbia; 5 Department of Gynecology, Oncology Institute of Vojvodina, 21204 Sremska Kamenica, Serbia; kojic.marina@onk.ns.ac.rs

**Keywords:** rectal cancer (RC), extramural venous invasion (EMVI), predictive medicine

## Abstract

**Simple Summary:**

Rectal cancer (RC) is the third most common gastrointestinal malignancy, with a rising incidence, particularly among the population under 50 years old. Extramural venous invasion (EMVI) is defined as the presence of malignant cells in veins beyond the muscularis propria near the primary colorectal tumor. The aim of our retrospective study was to assess the prognostic value of separate pathological EMVI reporting in operative RC samples and to determine its relationship with standard pathohistological and surgical parameters among a selected cohort of RC patients from our institution. Finally, EMVI is proved not only to be a feature of aggressive tumor behavior, but also as a separate and independent parameter in patients with rectal cancer. The results we obtained strongly suggest the importance of separately reporting extramural vascular invasion (EMVI) from lymphovascular invasion (LVI) in daily practice, as well as that EMVI could be a good addition to TNM staging.

**Abstract:**

Background/Objectives: Vascular invasion, especially extramural vascular invasion (EMVI), has emerged as a prognostic parameter for rectal cancer (RC) in recent years. Prediction of recurrence and metastasis development poses a significant challenge for oncologists, who need markers for prediction of adverse outcome. The aim of this study was to examine the prognostic significance of pathohistologically detected EMVI in untreated rectal cancer and its implications in separate reporting. Methods: We examined 100 untreated RC patients who underwent curative resection from January 2016 to June 2018 with a follow-up of 5 years. Patients were divided into equal EMVI− and EMVI+ groups based on histological re-examination of H&E-stained postoperative surgical samples. Results: The presence of EMVI within the selected cohort was significantly associated with female gender, T3/T4 and N1/N2 post-operative stages, positive lymph nodes, lymph node ratio LNR2 and LNR3 groups, abundant tumor-infiltrating lymphocytes, positive lympho-vascular invasion (LVI), perineural (PNI), and circumferential resection margin (CRM) (*p* < 0.05 in all tests). Within EMVI+ patients, local recurrences and/or metastases and death outcomes were more frequent events (*p* = 0.029 and *p* = 0.035, respectively), while survival analyses revealed that EMVI+ patients had significantly shorter overall survival (OS, *p* = 0.040) and disease-free survival (DFS, *p* = 0.028). Concerning LVI, differences in OS between LVI+ and LVI− patients were not statistically significant (*p* = 0.068), while LVI+ patients had significantly shorter DFS (*p* = 0.024). Moreover, univariate COX regression analysis demonstrated the negative impact of EMVI on OS (HR: 2.053, 95% CI: 1.015–4.152; *p* = 0.045) and DFS (HR: 2.106, 95% CI: 1.066–4.870; *p* = 0.038), which was not the case for LVI + RC patients. Conclusions: The obtained results strongly suggest the significance of separate reporting of EMVI from lympho-vascular invasion, as it is potentially a surrogate marker for adverse prognosis and outcome.

## 1. Introduction

Rectal cancer (RC) is the third most common gastrointestinal malignancy, with a rising incidence, particularly among the population under 50 years old [1,2]. Advances in managing rectal cancer have led to the development of multimodal treatment strategies, including the combined use of preoperative chemo/radiotherapy followed by surgery with or without adjuvant chemotherapy for stage II and stage III RC patients [3]. Mesorectal excision is the primary treatment option for localized rectal cancer [4]. However, treating stage II RC patients remains a significant clinical challenge due to the higher risk of local and systemic recurrence. Although criteria such as clinicopathologic features, patient age, and specific tumor characteristics provide a framework for using adjuvant treatment in stage II rectal cancer, the decision is complex and should be individualized [5,6,7,8]. Further research is needed to refine these criteria and improve treatment outcomes.

The progression and spread of the primary tumor are crucial factors for predicting the prognosis. In addition to TNM stage, several other prognostic and predictive factors have been reported by pathologists, including tumor perforation, perineural invasion, tumor deposits, and budding [9]. Although lymphovascular invasion (LVI) has previously been reported by pathologists, separate vascular invasion, especially extramural vascular invasion, has been proven to be an important factor of adverse prognosis in colorectal cancer [9]. Extramural venous invasion (EMVI) is defined as the presence of malignant cells in veins beyond the muscularis propria near the primary colorectal tumor [10]. Invasion of extramural veins allows tumor cells to travel through the portal or systemic circulation, which is a critical step in the metastatic process [11]. Pathologically detected EMVI in rectal carcinoma is associated with a higher incidence of local and distant metastases, as well as worse overall survival [12,13,14]. Considering recent developments that have enabled the analysis of EMVI on preoperative MRI of the pelvis, the documented presence of EMVI can be a critical factor in stratifying and treating patients with locally advanced rectal cancer (LARC) [15]. Radiomic models that include EMVI status, alongside other clinical factors, have been shown to effectively predict disease-free survival (DFS) in LARC patients [16,17,18]. These models achieve high predictive performance, indicating the utility of integrating EMVI with other prognostic markers.

While EMVI is a significant prognostic marker, its role should be considered alongside other clinical and pathological factors. The College of American Pathologists (CAP) emphasizes the importance of individual reporting of vascular invasion in the routine work of oncological surgical specimens [19]. This organization especially highlights the importance of detecting EMVI as an independent factor of poor prognosis and increased risk of liver metastases, while the relevance of intramural venous and lymphatic invasion is less clear and therefore neglected in routine work. In addition, the reporting of extra- and intramural vascular invasion presence is recommended but not a mandatory element of the pathohistological report [20]. While the detection and reporting of EMVI provide significant prognostic and therapeutic insights, challenges remain in standardizing imaging techniques and interpretation across different clinical settings.

In this context, we performed a retrospective study to assess the prognostic value of separate pathological EMVI reporting in operative RC samples and to determine its relationship with standard pathohistological and surgical parameters among a selected cohort of RC patients from our institution.

## 2. Materials and Methods

### 2.1. Patient Characteristics

In this retrospective study, we examined the clinical database for patients diagnosed with rectal cancer, confirmed by pathohistological findings. A total of 100 patients underwent curative resection for rectal cancer between January 2016 and June 2018 at our institute. The exclusion criteria for patient selection were as follows: (1) patients who had not undergone previous surgical resection for colorectal cancer; (2) patients who had received neoadjuvant therapy, as preoperative treatment may impact EMVI status; (3) patients with synchronous or metachronous metastatic colorectal cancer; and (4) patients with another existing malignant disease. The disease stage was determined based on the eighth edition of the American Joint Committee on Cancer (AJCC) Cancer Staging Manual (14). The study was conducted in accordance with the Declaration of Helsinki and approved by the Ethics Committee of the Oncology Institute of Vojvodina (protocol code 4/23/1-1819, date of approval 17 May 2023).

### 2.2. Tumor Characteristics

The data regarding tumor location and the type of surgery performed were gathered from the patients’ medical history. The information collected includes TNM classification, disease stage, number of resected lymph nodes, number of metastatic-positive lymph nodes (PLNs), lymph node ratio (LNR), tumor deposit, histological grade (well-, moderately, poorly differentiated tumors), circumferential resection margin (CRM), perineural (PNI), lymphovascular invasion (LVI), tumor-infiltrating lymphocytes (TILs), and mucosal component of the tumor. The lymph node ratio is calculated by dividing the number of metastatic nodes to retrieved lymph nodes. Patients were divided into three groups: LNR 1 (0–0.19), LNR2 (0.20–0.39), and LNR3 (˃0.40).

### 2.3. Follow-Up

Patients underwent post-operative follow-up, including CT scans every 6 months for the first 2 years and then yearly scans for 5 years. Additionally, all patients had a colonoscopy performed 6 months after the surgery, with the frequency of subsequent endoscopic assessments determined by the pathology encountered. Disease-free survival (DFS) was measured from the date of surgery to the date of pelvic recurrence and/or distant disease. Overall survival (OS) was calculated from the date of diagnosis to the date of death from any cause. Disease progression was defined as the occurrence of local recurrence or distant metastasis. Clinical and pathological factors were compared to assess their impact on local recurrence, distant metastasis, DFS, and OS.

### 2.4. Pathological Analysis

Pathohistological slides of all included patient samples were re-examined by a pathologist who determined two groups of patients: with and without extramural venous invasion. Re-evaluation was performed on standard H&E-stained original postoperative sample slides based on localization and the size of the vessel. No additional staining was performed. In arbitrary cases, two board-certified pathologists reexamined the slides. Re-evaluation was conducted in order to select patients with just extramural venous invasion opposite lymphovascular invasion or just vascular invasion, as stated in prior pathohistological reports, according to the former protocols. The number of re-examined slides per patient was between 3 and 10.

### 2.5. Statistical Analysis

For contingency variables, the χ^2^-test or Fisher’s exact two-tailed test was used when the expected frequencies were lower than five. For continuous variables, the Student’s *t*-test was used. Continuous variables were expressed as mean ± standard deviation (X ± SD), while categorical variables were presented by number of cases (percentage). All variables that showed a significant correlation with death outcome and relapse of disease (*p* < 0.05) were analyzed using the Cox hazard ratio (Cox HR) model, which was used for both univariate and multivariate regression analysis. The variables that showed significant differences (*p* < 0.05) in univariate analysis were selected for multivariate regression analysis to assess predictors of OS and DFS. Overall and disease-free survival distributions were estimated by the Kaplan–Meier method, and differences were evaluated by the log-rank test. A *p*-value less than 0.05 was considered statistically significant in all tests. All statistical analyses were performed using the Sigma Plot 14.0 licensed statistical analysis software package.

## 3. Results

### 3.1. Patient Characteristics

This retrospective study included 100 RC patients who met the inclusion criteria, whose average age was 64.6 ± 9.6 years (range 40–83). The group consisted of 62 (62%) male and 38 (38%) female patients, whose average age did not differ significantly (65.1 ± 9.4 vs. 63.8 ± 9.9 years, *p* = 0.528). Lymphovascular invasion (LVI) was detected in 52/100 (52%) tumor samples, while in 18/100 (18%) cases, perineural invasion (PNI) was observed. According to our criteria of LNR classification, 79/100 (79%) cases were classified as LNR1 and 10/100 (10%) as LNR2, and 11/100 (11%) cases were included in the LNR3 group. After surgery was performed, 61/100 (61%) RC patients received adjuvant treatment.

### 3.2. Association of EMVI Status with Clinico-Pathological Parameters

**Figure 1:**
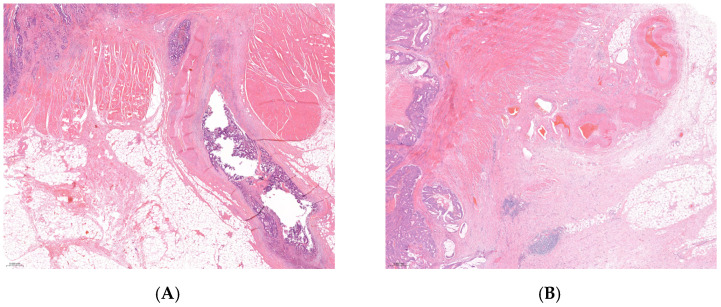
EMVI in rectal cancer. (**A**) Tumor cells present inside the lumens of venous spaces beyond the wall of the rectum and infiltrative borders of the tumor in T3-stage rectal cancer, H&E, 2.2× magnification. (**B**) Absence of tumor cells in venous spaces is considered to be EMVI negative in T2 rectal cancer, H&E, 2.2× magnification.

Out of the 100 selected RC patients, there were 50 EMVI+ and 50 EMVI− cases (Figure 1). Clinical and pathohistological characteristics of patients and tumors in relation to EMVI status are presented in Table 1. We should note that among EMVI-positive patients (EMVI+), 46/50 (92%) were in the NO pre-operative stage, 46 out of 50 (92%) were treated with sphincter-preserving surgery, and 47/50 (94%) EMVI+ patients received adjuvant treatment. The presence of EMVI within the selected cohort was significantly associated with female gender (*p* = 0.039), T3/T4 post-operative stages (*p* < 0.001), N1/N2 post-operative stages (*p* < 0.001), positive lymph nodes (PLN ˃ 3, *p* < 0.001), the LNR2 and LNR3 groups (*p* < 0.001), and abundant tumor-infiltrating lymphocytes (TILs) (*p* = 0.044). According to the binary classified TNM stage, there were significantly more EMVI+ cases among TNMIII/IV tumor stages than within TNMI/II (86.7% vs. 20%, *p* < 0.001). A significant association was also observed between the presence of EMVI and positive LVI, PNI, and CRM (*p* < 0.05 in all tests), while adjuvant therapy was more frequently applied to EMVI+ patients than to EMVI− patients (70.5% vs. 29.5%, *p* < 0.001).

### 3.3. Relapse of Disease and Death Outcome in RC Patients

In this study, the median follow-up period after surgery was 56 (range 12–76) months. The median survival without recurrence of disease was 52 (range 4–76) months. Out of the total RC patients, 66 (66%) were still alive during the follow-up period, while 30 (30%) had verified recurrence of disease. Clinical and pathohistological features related to the outcome and relapse of disease are listed in Table 2 and Table 3, respectively. As expected, advanced TNM stages, positive lymph nodes (PLN ˃ 3), and high LNR were significantly associated with more common death outcomes and relapse of disease (*p* < 0.05 in all tests). Considering EMVI status, there were more EMVI+ patients with local recurrences and/or metastases (44% vs. 24%) and death outcomes recorded (40% vs. 20%) than EMVI− cases (*p* = 0.035 and *p* = 0.029, respectively). The presence of LVI was also associated with relapse of disease (*p* = 0.018), while we noted a statistical trend toward the relationship between LVI+ and death outcome (*p* = 0.068). In addition, adjuvant treatment was significantly related to more common death and relapse events (*p* = 0.023 and *p* < 0.001, respectively).

### 3.4. Overall and Disease-Free Survival of RC Patients

Results of univariate and multivariate Cox regression analysis for the overall survival period are listed in Table 3. Regarding the clinicopathological parameters, univariate analysis showed that OS of RC patients was significantly associated with N1 preoperative stage (*p* = 0.003), PLN ˃ 3 (*p* < 0.001), LNR2 and LNR3 (*p* = 0.038 and *p* < 0.001, respectively), TNMIII/IV stages (*p* = 0.023), the presence of EMVI (*p* = 0.045), and positive CRM (*p* = 0.030). Analysis of EMVI status in relation to OS by univariate COX regression revealed that EMVI+ patients had a 2.053 times higher risk of death during the period of postoperative follow-up (95% CI: 1.015–4.152; *p* = 0.045). The remaining analyzed risk parameters—T and N pathological stage, and LVI and PNI status—did not show a statistically significant association with overall survival time by univariate analysis (*p* > 0.05), while received adjuvant therapy almost reached statistical significance (*p* = 0.05). After performing multivariate Cox regression, none of the included risk parameters retained statistical significance (*p* ˃ 0.05).

Univariate and multivariate Cox regression analyses for disease-free survival are summarized in Table 4. Using univariate Cox regression for DFS, a statistically significant relationship was found with pre-operative N (*p* < 0.001), PLN ˃ 3 (*p* < 0.001), LNR3 (*p* < 0.001), TNM III/IV stage (*p* = 0.017), and positive EMVI and CRM (*p* = 0.038 and *p* = 0.013, respectively). Regarding EMVI status, univariate analysis revealed that EMVI+ patients had a 2.106 times higher risk of disease recurrence than EMVI− patients (95% CI: 1.066–4.870; *p* = 0.038). After adjustment in multivariate analysis, N1 pre-operative stage (HR: 4.632, 95% CI: 1.255–17.100; *p* = 0.021) and positive CRM (HR: 3.331, 95% CI: 1.059–10.480; *p* = 0.040) were obtained as an independent prognostic factor of survival time without relapse in RC patients, while statistical significance for EMVI+ was lost in multivariate regression analysis (*p* = 0.996) (Table 5).

### 3.5. Comparison of Prognostic Significance of EMVI and LVI Status

**Figure 2:**
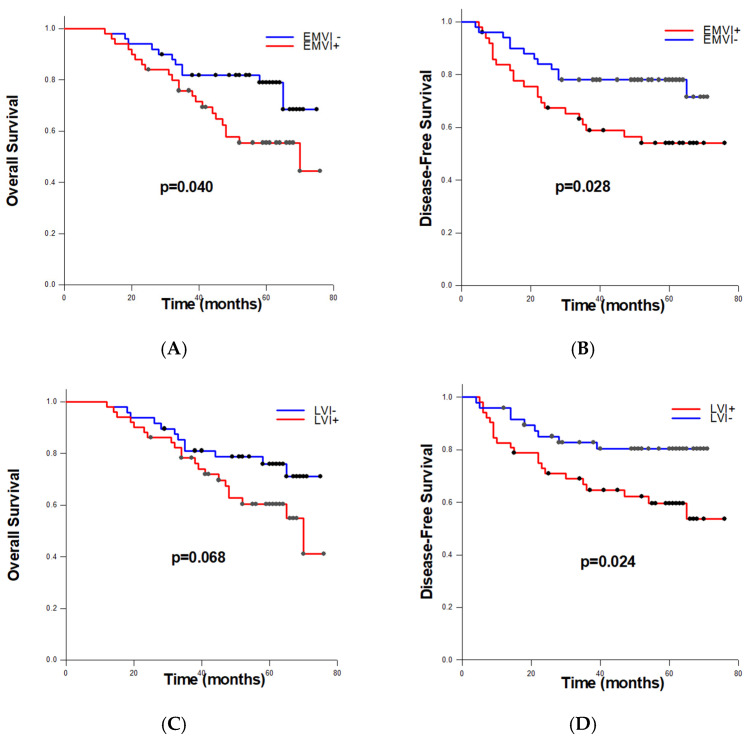
Kaplan–Meier survival curves for overall (**A**,**C**) and disease-free survival (**B**,**D**), stratified by extramural venous invasion (**A**,**B**) and lymphovascular invasion (**C**,**D**).

To estimate the obtained differences in overall and disease-free survival among RC patients according to EMVI and LVI status, we performed log-rank tests. Patients with detected EMVI had significantly shorter average OS (56.230 ± 3.350 months) compared to patients without EMVI (64.640 ± 2.845 months) (*p* = 0.040) (Figure 2A). Moreover, among EMVI+ cases, significantly shorter average DFS was recorded than within EMVI− cases (52.162 ± 4.319 vs. 61.338 ± 3.041 months, *p* = 0.028) (Figure 2B). Concerning lymphovascular invasion, differences in overall survival between LVI+ and LVI− patients were not statistically significant (*p* = 0.068), while patients identified as LVI positive had significantly shorter disease-free survival (*p* = 0.024) (Figure 2C,D).

### 3.6. Comparison of Prognostic Significance of EMVI and N Status

**Figure 3:**
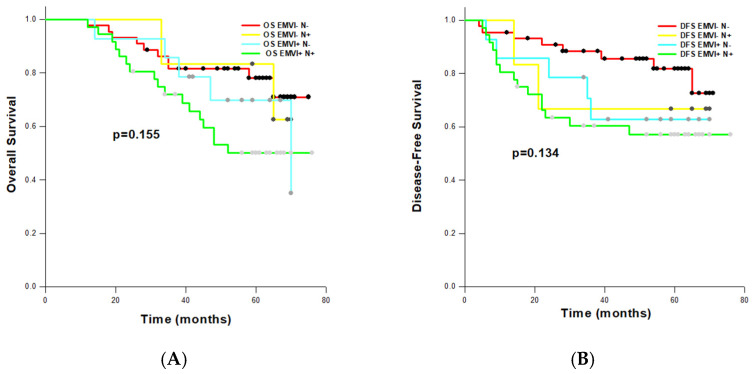
Kaplan–Meier survival curves for overall (**A**) and disease-free survival (**B**), stratified by extramural venous invasion and nodal status.

To assess the combined impact of EMVI and pathological nodal (N) status on OS and DFS, we defined four categories according to the EMVI and N status: EMVI−/N−, EMVI+/N−, EMVI−/N+, and EMVI+/N−. On univariate analysis, EMVI+/N+ combination was a significant factor for reduced OS (HR: 2.369, 95% CI: 1.084–5.178; *p* = 0.031) and DFS (HR: 2.699, 95% CI: 1.143–6.375; *p* = 0.024). As expected, EMVI+/N+ cases had the shortest OS (54.452 ± 4.080 months) and DFS (50.624 ± 5.267 months) estimated by the Log-rank tests (Figure 3A,B). We also noted that EMVI+/N− patients had worse overall and disease-free survival (OS: 59.135 ± 5.403; DFS: 52.364–7.322 months) than EMVI−/N+ patients (OS: 62.792 ± 7.797; DFS: 52.500 ± 14.336 months), although the observed differences were not statistically significant (OS: *p* = 0.155; DFS: *p* = 0.134).

## 4. Discussion

Venous invasion is routinely evaluated in colorectal cancer in daily clinical practice and is classified as intramural and extramural. The anatomic location of the invaded vessel can be important for predicting the prognosis of patients with rectal cancer [9,21]. These tumors have an increased potential for vascular seeding. Since the tumor is aggressive enough to invade blood vessels directly, it makes sense that these patients are at higher risk of having occult disease [22]. Within this study, extramural venous invasion was observed as a potentially negative predictor of rectal cancer outcome. Finally, extramural venous invasion (EMVI) is proven to be not only a feature of aggressive tumor behavior but also a separate and independent parameter in patients with rectal cancer. This is in accordance with multiple studies demonstrating its association with poorer patient outcomes [23].

The current study proved that EMVI is significantly associated with already established parameters of worse prognosis, including T3/T4 pathological stages (*p* < 0.001), N1/N2 pathological stages (*p* < 0.001), positive lymph nodes (PLN > 3, *p* < 0.001), LNR2 and LNR3 (*p* < 0.001), as well as abundant TIL (*p* = 0.004) and positive LVI, PNI, and CRM (*p* < 0.05 in all tests), which is in agreement with the results of multiple studies [12,22,24].

While it has been well established that patients with stage III colorectal cancer should be offered adjuvant chemotherapy [25,26], the guidelines for stage II colorectal cancer are not as clear. The current guidelines from the Association of Coloproctology of Great Britain & Ireland (ACGBI), as well as the European Society for Medical Oncology, suggest that patients with high-risk stage II disease, of which EMVI is considered one of the high-risk factors, should be considered for adjuvant chemotherapy [12,27,28]. According to this study, adjuvant therapy was more frequently applied on EMVI+ patients than on EMVI− patients (*p* < 0.001), which is consistent with the finding by Mc Entee et al., who emphasize that the presence of EMVI should be strongly considered as an indication for adjuvant therapy [24]. This is consistent with the findings of McClelland and colleagues, who also claim that EMVI+ patients in stage II may benefit from and should be strongly considered for adjuvant chemotherapy [12].

Extramural venous invasion (EMVI) has been identified as a strong and independent predictor of poor prognosis and increased risk of disease recurrence [12,22,24,28]. In our study, more EMVI+ patients experienced local recurrences and/or metastases than EMVI- patients (*p* = 0.035), which is consistent with previous research findings [10,22,24,28]. Additionally, a higher number of EMVI+ patients had recorded instances of death compared to EMVI− patients (*p* = 0.029), which also aligns with other studies [22,24].

The main finding of this study is that patients with detected EMVI had significantly shorter average overall survival (OS) and disease-free survival (DFS) compared to EMVI− patients, whether they had stage II or stage III rectal cancer (*p* = 0.040 and *p* = 0.028, respectively), which is consistent with other research findings [12,21,22,24,28]. Furthermore, this study revealed that differences in OS between LVI+ and LVI– patients were not statistically significant (*p* = 0.068), suggesting the potential superiority of EMVI as a separate and independent negative predictor of disease. Moreover, univariate COX regression analysis demonstrated the negative impact of EMVI on OS (HR: 2.053, 95% CI: 1.015–4.152; *p* = 0.045) and DFS (HR: 2.106, 95% CI: 1.066–4.870; *p* = 0.038), which was not the case for LVI+ RC patients. This result aligns with previous recommendations suggesting that lymphatic and vascular invasion, especially EMVI, should be separately reported [10,29,30,31] due to their distinct spread pathways and impacts on tumor aggressiveness [9].

For further discrimination among the prognostic significance of EMVI and lymphatic invasion (LI) as distinct pathways of invasion in RC, we used N stadium as a surrogate marker for LI and divided patients according to EMVI and N status in four categories. The EMVI+/N− patient category recorded worse OS and DFS than in the EMVI−/N+ category of patients, suggesting the dominance of EMVI status in prognostic stratification of patients, although the observed differences were not statistically significant (*p* = 0.155 and *p* = 0.134, respectively). Similar importance of EMVI status was observed in the study by Chand et al. [28], while other authors provided evidence that the accuracy of nodal staging is limited and that it has not consistently demonstrated prognostic importance in rectal cancer [32]. Although our results of univariate COX regression analysis clearly demonstrated the negative impact of EMVI on OS and DFS, after adjusting in multivariate analysis, the statistical significance for EMVI+ was lost, which is not in accordance with other studies [22,33,34]. Thus, the exact role of postoperative detected EMVI should be re-evaluated with a larger number of patients included in the study.

Methods for evaluating EMVI include preoperative radiologic and postoperative pathologic examinations [16]. Recent advances in MRI mean it is possible to assess EMVI in pre-operative investigations for rectal cancer [35]. EMVI assessed by MRI has been associated with an increased risk of local and systemic disease recurrence and diminished survival [18,23,35,36]. MRI detection of EMVI (mrEMVI) has been shown to be accurate and correlate highly with pathology for patients who have undergone primary surgery [21,23]. The College of American Pathologists recommends recording the status of extramural vascular invasion during routine pathologic examination in rectal cancer patients [19]. A pathological finding of EMVI should prompt strong consideration of adjuvant therapy for patients who have undergone a curative colorectal cancer resection [22,24]. In daily practice, it is not common for EMVI to be reported as part of a routine pathologic examination. Depending on the institutional practice, sometimes it is reported as vascular invasion and sometimes as part of LVI. However, the results of this study show that it makes sense to consider EMVI separately from LVI, as well as that EMVI could be a good addition to TNM staging. In addition, EMVI would have even greater prognostic potential if routinely observed on preoperative pelvic MRI.

## 5. Conclusions

The results we obtained strongly suggest the importance of separately reporting extramural vascular invasion (EMVI) from lymphovascular invasion. EMVI could potentially serve as a surrogate marker for adverse prognosis and outcome. Our findings indicate that patients with detected EMVI had significantly shorter average overall survival (OS) and disease-free survival (DFS) compared to EMVI− patients, even in the lower stages of rectal cancer. Although there was no statistical significance of OS and DFS between EMVI+/N− and EMVI−/N+ categories of patients, comparison of EMVI to other aforementioned variables demonstrated statistical significance, which still makes EMVI a superior parameter in patients with rectal cancer and confirms the importance of its separate reporting in daily practice. Therefore, we need to pay closer attention to detecting and reporting EMVI during both pathological and radiological examinations in order to improve RC patient stratification and disease outcome prediction.

## Figures and Tables

**Table 1:** Association of EMVI status with clinico-pathological parameters.

Parameters	EMVI Status		*p* *
	EMVI+ N (%)	EMVI− N (%)	
**Age (years)**	63.24 ± 9.87	65.94 ± 9.148	0.159
Gender			
Male	26/62 (41.9)	36/62 (58.1)	**0.039**
Female	24/38 (63.2)	14/38 (36.8)	
Preoperative T-stage			
T1/T2	22/52 (42.3)	30/52 (57.7)	0.109
T3	28/48 (58.3)	20/48 (41.7)	
Preoperative N-stage			
N0	46/94 (48.9)	48/94 (51.1)	0.678
N1	4/6 (66.7)	2/6 (33.3)	
Localization			
0–5 cm	10/23 (43.5)	13/23 (56.5)	0.775
5–10 cm	15/29 (51.7)	14/29 (48.3)	
10–15 cm	25/48 (52.1)	23/48 (47.9)	
Sphincter-preserving surgery			
Yes	46/92 (50)	46/92 (50)	1.000
No	4/8 (50)	4/8 (50)	
Pathological T-stage			
T1/T2	3/24 (12.5)	21/24 (87.5)	**<0.001**
T3/T4	47/76 (61.8)	29/76 (38.2)	
Pathological N-stage			
N0	14/58 (24.1)	44/58 (75.9)	**<0.001**
N1/N2	36/42 (85.7)	6/42 (14.3)	
PLN			
≤3	29/79 (36.7)	50/79 (63.3)	**<0.001**
˃3	21/21 (100)	0/21 (0)	
LNR			
LNR1 (0–0.19)	30/79 (38.0)	49/79 (62.0)	**<0.001**
LNR2 (0.20–0.39)	9/10 (90)	1/10 (10)	
LNR3 (˃0.40)	11/11 (100)	0/11 (0)	
TNM stage			
I/II	11/55 (20)	44/55 (80)	**<0.001**
III/IV	39/45 (86.7)	6/45 (13.3)	
Gradus			
G1	4/7 (57.1)	3/7 (42.9)	0.189
G2	36/79 (45.6)	43/79 (54.4)	
G3	10/14 (71.4)	4/14 (28.6)	
Mucosal component of the tumor			
Yes	45/92 (48.9)	47/92 (51.1)	0.715
No	5/8 (62.5)	3/8 (37.5)	
TIL			
Scarce	24/45 (53.3)	21/45 (46.7)	**0.044**
Moderate	6/22 (27.3)	16/22 (72.7)	
Abundant	20/33 (60.6)	13/33 (39.4)	
LVI			
Positive	45/52 (86.5)	7/52 (13.5)	**<0.001**
Negative	5/48 (10.4)	43/48 (89.6)	
PNI			
Positive	15/18 (83.3)	3/18 (16.7)	**0.002**
Negative	35/82 (42.7)	47/82 (57.3)	
CRM			
Positive	9/9 (100)	0/9 (0)	**0.003**
Negative	41/91 (45.1)	50/91 (54.9)	
Adjuvant treatment			
Yes	43/61 (70.5)	18/61 (29.5)	**<0.001**
No	7/39 (17.9)	32/39 (82.1)	

* All *p*-values obtained using the χ^2^ test. N—number of patients; EMVI—extramural venous invasion; PLN—number of metastatic-positive lymph nodes; LNR—lymph node ratio; TIL—tumor-infiltrating lymphocytes; LVI—lymphovascular invasion; PNI—perineural invasion; CRM—circumferential resection margin.

**Table 2:** Clinical characteristics in relation to death outcome and disease progression.

Parameters	Death Outcome		p *	Relapse of Disease		*p* *
	YES n (%)	NO n (%)		YES n (%)	NO n (%)	
Age (years)	64.00 ± 10.86	64.89 ± 8.89	0.660	61.30 ± 9.30	66.0 ± 9.39	**0.024**
Gender						
Male	22/62 (35.5)	40/62 (64.5)	0.689	18/62 (29)	44/62 (71)	0.787
Female	12/38 (31.6)	26/38 (68.4)		12/38 (31.6)	26/38 (68.4)	
Preoperative T-stage						
T1/T2	16/52 (30.8)	36/52 (69.2)	0.478	12/52 (23.1)	40/52 (76.9)	0.116
T3	18/48 (37.5)	30/48 (62.5)		18/48 (37.5)	30/48 (62.5)	
Preoperative N-stage						
N0	28/94 (29.8)	66/94 (70.2)	**0.001**	24/94 (25.5)	70/94 (74.5)	**<0.001**
N1	6/6 (100)	0/6 (0)		6/6 (100)	0/6 (0)	
Localization						
0–5 cm	10/23 (43.5)	13/23 (56.5)	0.491	8/23 (34.8)	15/23 (65.2)	0.577
5–10 cm	10/29 (34.5)	19/29 (65.5)		10/29 (34.5)	19/29 (65.5)	
10–15 cm	14/48 (29.2)	34/48 (70.8)		12/48 (25)	36/48 (75)	
Sphincter-preserving surgery						
Yes	31/92 (33.7)	61/92 (66.3)	1.000	27/92 (29.3)	65/92 (70.7)	0.694
No	3/8 (37.5)	5/8 (62.5)		3/8 (37.5)	5/8 (62.5)	
Adjuvant treatment						
Yes	26/61 (42.6)	35/61 (57.4)	**0.023**	26/61 (42.6)	35/61 (57.4)	**<0.001**
No	8/39 (20.5)	31/39 (79.5)		4/39 (10.3)	35/39 (89.7)	

* All *p*-values obtained using the χ^2^ test. n—Number of patients.

**Table 3:** Pathohistological characteristics in relation to death outcome and disease progression.

Parameters	Death Outcome		p *	Relapse of Disease		p *
	YES n (%)	NO n (%)		YES n (%)	NO n (%)	
Pathological T-stage						
T1/T2	4/24 (16.7)	20/24 (83.3)	**0.040**	3/24 (12.5)	21/24 (87.5)	**0.032**
T3/T4	30/76 (39.5)	46/76 (60.5)		27/76 (35.5)	49/76 (64.5)	
Pathological N-stage						
N0	15/58 (25.9)	43/58 (74.1)	**0.044**	13/58 (22.4)	45/58 (77.6)	0.052
N1/N2	19/42 (45.2)	23/42 (54.8)		17/42 (40.5)	25/52 (48.1)	
PLN						
≤3	20/79 (25.3)	59/79 (74.7)	**0.004**	18/79 (22.8)	61/79 (77.2)	**0.020**
˃3	14/21 (66.7)	7/21 (33.3)		12/21 (57.1)	9/21 (42.9)	
LNR						
LNR1 (0–0.19)	20/79 (25.3)	59/79 (74.7)	**<0.001**	18/79 (22.8)	61/79 (77.2)	**0.002**
LNR2 (0.20–0.39)	5/10 (50)	5/10 (50)		4/10 (40)	6/10 (60)	
LNR3 (˃0.40)	9/11 (81.8)	2/11 (18.2)		8/11 (72.7)	3/11 (27.3)	
TNM stage						
I/II	13/55 (23.6)	42/55 (76.4)	**0.016**	11/55 (20)	44/55 (80)	**0.016**
III/IV	21/45 (46.7)	24/45 (53.3)		19/45 (42.2)	26/45 (57.8)	
Grade						
G1	2/7 (28.6)	5/7 (71.4)	0.143	2/7 (28.6)	5/7 (71.4)	0.057
G2	24/79 (30.4)	55/79 (69.6)		20/79 (25.3)	59/79 (74.7)	
G3	8/14 (57.1)	6/14 (42.9)		8/14 (57.1)	6/14 (42.9)	
Mucosal component of the tumor						
Yes	4/8 (50)	4/8 (50)	0.439	3/8 (37.5)	5/8 (62.5)	0.694
No	30/92 (32.6)	62/92 (67.4)		27/92 (29.3)	65/92 (70.7)	
TIL						
Scarce	18/45 (40)	27/45 (60)	0.373	15/45 (33.3)	30/45 (66.7)	0.391
Moderate	5/22 (22.7)	17/22 (77.3)		4/22 (18.2)	18/22 (81.8)	
Abundant	11/33 (33.3)	22/33 (66.7)		11/33 (33.3)	22/33 (66.7)	
LVI						
Positive	22/52 (42.3)	30/52 (57.7)	0.068	21/52 (40.4)	31/52 (59.6)	**0.018**
Negative	12/48 (25)	36/48 (75)		9/48 (18.7)	39/48 (81.3)	
EMVI						
Positive	22/50 (44)	28/50 (56)	**0.035**	20/50 (40)	30/50 (60)	**0.029**
Negative	12/50 (24)	38/50 (76)		10/50 (20)	40/50 (80)	
PNI						
Positive	10/18 (55.6)	8/18 (44.4)	**0.033**	7/18 (38.9)	11/18 (61.1)	0.363
Negative	24/82 (29.3)	58/82 (70.7)		23/82 (28)	59/82 (72)	
CRM						
Positive	6/9 (66.7)	3/9 (33.3)	0.059	5/9 (55.6)	4/9 (44.4)	0.123
Negative	28/91 (30.8)	63/91 (69.2)		25/91 (27.5)	66/91 (72.5)	

* All *p*-values obtained using the χ^2^ test. n—Number of patients; EMVI—extramural venous invasion; PLN—number of metastatic-positive lymph nodes; LNR—lymph node ratio; TIL—tumor-infiltrating lymphocytes; LVI—lymphovascular invasion; PNI—perineural invasion; CRM—circumferential resection margin.

**Table 4:** Univariate and multivariate Cox regression analysis for overall survival.

Parameters	Univariate		*p*	Multivariate		*p*
	HR	95% CI		HR	95% CI	
N1 preoperative stage	3.880	1.592–9.455	0.003	2.854	0.800–10.180	0.106
T0 pathological stage	0.389	0.137–1.108	0.077	/	/	/
N1 pathological stage	1.898	0.963–3.737	0.064	/	/	/
PLN (n ˃ 3)	3.756	1.875–7.526	<0.001	1.327	0.290–6.063	0.715
LNR2	2.845	1.062–7.626	0.038	2.442	0.576–10.350	0.226
LNR3	4.691	2.096–10.500	<0.001	2.190	0.426–11.270	0.348
TNM III/IV	2.230	1.116–4.455	0.023	0.622	0.169–2.279	0.474
LVI positive	1.914	0.946–3.871	0.071	/	/	/
EMVI positive	2.053	1.015–4.152	0.045	1.127	0.410–3.095	0.817
PNI positive	1.988	0.948–4.160	0.068	/	/	/
CRM positive	2.654	1.096–6.427	0.030	2.271	0.748–6.899	0.148
Adjuvant treatment	2.212	1.001–4.888	0.050	1.416	0.460–4.395	0.544

HR—hazard ratio; CI—confidence interval; EMVI—extramural venous invasion; PLN—number of metastatic-positive lymph nodes; LNR—lymph node ratio; LVI—lymphovascular invasion; PNI—perineural invasion; CRM—circumferential resection margin.

**Table 5:** Univariate and multivariate Cox regression analysis for disease-free survival.

Parameters	Univariate		*p*	Multivariate		*p*
	HR	95% CI		HR	95% CI	
N1 preoperative stage	6.009	2.402–15.030	<0.001	4.632	1.255–17.100	**0.021**
T0 pathological stage	0.372	0.131–1.057	0.064	/	/	/
N1 pathological stage	2.026	1.028–3.993	0.041	0.275	0.045–1.680	0.163
PLN (n ˃ 3)	3.673	1.848–7.301	<0.001	1.568	0.330–6.760	0.572
LNR2	2.509	0.941–6.694	0.066	/	/	/
LNR3	4.910	2.207–10.920	<0.001	1.915	0.369–9.926	0.575
TNM III/IV	2.325	1.162–4.649	0.017	2.047	0.259–16.170	0.497
LVI positive	1.881	0.930–3.802	0.079	/	/	/
EMVI positive	2.106	1.042–4.257	0.038	0.995	0.329–3.003	0.993
PNI positive	2.012	0.962–4.207	0.063	/	/	/
CRM positive	3.069	1.267–7.434	0.013	3.331	1.059–10.480	**0.040**
Adjuvant treatment	2.294	1.038–5.070	0.040	1.483	0.483–1.687	0.491

HR—hazard ratio; CI—confidence interval; EMVI—extramural venous invasion; PLN—number of metastatic-positive lymph nodes; LNR—lymph node ratio; LVI—lymphovascular invasion; PNI—perineural invasion; CRM—circumferential resection margin.

## Data Availability

The raw data supporting the conclusions of this article will be made available by the authors on request.
